# Reinforcement of Peer-Coaching and Clinical Audit to Improve Implementation of Package for Essential Non-Communicable Diseases (PEN) in Nepal: A Pilot Implementation Study Method

**DOI:** 10.31729/jnma.9017

**Published:** 2025-05-31

**Authors:** Dipanker Prajapati, Sujata Sapkota, Punya Shori Suwal, Anu Aryal, Rashmi Maharjan, Sudip Ale Magar, Kamala Rana Magar, Kabita Poudel, Prajjwal Pyakurel, Abhinav Vaidya, Archana Shrestha

**Affiliations:** 1 Shahid Gangalal National Heart Centre, Bansbari, Kathmandu, Nepal; 2 National Academy of Medical Sciences, Mahaboudha, Kathmandu, Nepal; 3 Manmohan Memorial Institute of Health Sciences, Soaltemode, Kathmandu, Nepal; 4 Department of Public Health and Community Programs, Dhulikhel Hospital, Kathmandu University Hospital, Dhulikhel, Kavre, Nepal; 5 Central Institute of Science and Technology, Baneshwor, Kathmandu, Nepal; 6 Department of Health Policy and Management, University of California, Los Angeles, USA; 7 Department of Nursing and Midwifery, Kathmandu University School of Medical Sciences, Dhulikhel, Kavre, Nepal; 8 Department of Health Services, Ministry of Health and Population, Teku, Kathmandu, Nepal; 9 Pokhara Academy of Health Sciences, Ramghat, Pokhara, Nepal; 10 SAARC TB and HIV/AIDS Centre, Thimi, Bhaktapur, Nepal; 11 Kathmandu Medical College, Sinamangal, Kathmandu, Nepal

**Keywords:** *clinical audit*, *non-communicable diseases*, *package of essential non-communicable disease*, *team-based care*, *World Health Organization*

## Abstract

**Introduction:**

Nepal endorsed and implemented the WHO Package of Essential Non-communicable Disease Intervention. However, its implementation is far from satisfactory. We designed and implemented an intervention to reinforce peer coaching and clinical audit mechanisms in primary-level health facilities, and tested its feasibility and preliminary effectiveness. This paper details the methodology used in designing, implementing and assessing the intervention.

**Methods:**

The study adoptes a hybrid type II implementation trial design. The intervention assignment followed a non-blinded, two-arm, parallel randomized controlled trial design with a 1:1 allocation ratio. Seventeen primary-level public health facilities in Bhaktapur district with at least one trained staff were randomized. The clinical staff at the intervention health facilities received peer-coaching and clinical audit reinforcement, while the control group followed their usual practice. The study was conducted over a 12-month duration from Februay 2022, to January, 2023. A mixed-method approach, applying pre-post assessment and thematic analysis, to inform the intervention development and assess implementation outcomes and its effectiveness was adopted. The study was guided by the Proctor framework. Ethical clearance was obtained from the Nepal Health Research Council (Registraion number: 302/2021).

**Discussion:**

The tools and methods that guide intervention implementation and assessment have the potential to be replicated in various settings to design strategies to improve Package of Essential Non-communicable Disease Intervention adoption and sustainment.

**Trial Registration:**

This trial is registered with ClinicalTrials.gov (www.clinicaltrials.gov), Identifier: NCT05880784.

## INTRODUCTION

Nepal endorsed WHO Package of Essential Non-communicable Disease (PEN) interventions for early detection and effective management of Non-Communicable Diseases (NCDs).^1^ However, several factors including limited capacity of health institutions', shortage of human resources, logistics and poor documentation have hindered the effective implementation.^2-4^

PEN recommends a team-based care approach that includes utilizing peer-coaching and clinical audit mechanisms.^1^ It emphasizes peer coaching as a modality of team support and communication and clinical audit as a monitoring and evaluation step. Studies demonstrate these mechanisms as cost-effective measures to improve NCD care.^5-8^ Nonetheless, it is not known if and how these mechanisms are utilized in supporting PEN implementation in Nepal.

To reinforce peer-coaching and clinical audit practices and thereby improve PEN interventions implementation, we designed, implemented and assessed an intervention. Here, we detail the method, including development, implementation and assessment of the intervention. Study findings are beyond the scope of this paper.

## METHODS

### Study Design

A hybrid type II implementation trial design with two aims-a) assessing implementation outcomes based on Proctor's framework,^9^ including acceptability, adoption, feasibility, penetration, cost, and sustainability of the intervention, and b) assessing the effectiveness of the intervention was adopted. The intervention assignment followed a non-blinded, two-arm; parallel randomized controlled trial with a 1:1 allocation ratio. Health facilities were the unit of randomization, and the inclusion criteria for the study were government-run primary-level health facilities (primary health centers and municipal hospitals) in Bhaktapur district that have at least one PEN-trained staff. To ascertain the eligibility, we contacted each facility via telephone. Out of 21 government-run health facilities, 17 facilities met the eligibility criteria and were included in our study. The clinical staff at the intervention health facilities were the intervention targets and received the peer-coaching and clinical audit reinforcement intervention (detailed below), while the control group followed their usual practice. The study was conducted over a 12-month duration from February 2022 to January 2023. We used a mixed-method approach to inform the development of intervention and assess implementation outcomes using the Proctor framework.^9^ The reporting of the trial is guided by SPIRIT guidelines.^10^

### Study setting

We conducted the study in public primary health facilities (including health posts and municipality hospitals) in the Bhaktapur district, where the PEN program has been implemented since 2018. Bhaktapur district has four municipalities and a total population of 4,30,408. There are 21 public health facilities in Bhaktapur.^11^

### Ethics and consent process

The study is registered with ClinicalTrials.gov (Registration number: NCT05880784). Ethical clearance was obtained from the Nepal Health Research Council (Registration number: 302/2021) on 29 July 2021. Additionally, permission to conduct the study was obtained from the Health Office, Bhaktapur and all municipalities in Bhaktapur.

Participants were oriented about the project, its objectives and their role in the intervention. Written informed consent was obtained from all participants at each stage of intervention and data collection.

### Study team

The study team consisted of clinicians, pharmacist, nurses, public health professionals, community physicians, and academics/researchers. We recruited two research assistants (RA) who had bachelor's degrees in public health. As part of the research training, the RA attended a 4-day Training of Trainers workshop on WHO PEN Interventions conducted by the National Health Training Centre, Ministry of Health and Population, Nepal. Furthermore, they were trained on data collection tools/techniques, PEN evaluation forms, peer coaching, and clinical audits by research team leads. The role of the RA was to collect baseline and end-line data, support health facility teams in conducting peer coaching and clinic audits, including filling out evaluation forms for peer coaching sessions, and take notes during focus group discussions.

### Intervention development and implementation

Our intervention consisted of reinforcing peer coaching and clinical audit processes as indicated in the WHO PEN interventions guideline endorsed by the Nepal Government.^12^ Among the four protocols of the WHO PEN interventions, our study focused exclusively on 'PEN Protocol 1' (Prevention of Heart Attack, Strokes and Kidney Disease through Integrated Management of Diabetes and Hypertension).^12^

Intervention development and implementation process was conducted in two phases. The Preparatory Phase includes activities aimed at understanding the existing context to help inform the intervention development and delivery process. The Intervention Phase includes the process of intervention implementation and assessment of the implementation outcomes.

### Preparatory Phase

*Baseline assessment:* A baseline assessment was conducted with the primary objective of exploring the existing PEN Interventions implementation scenario and the status of peer-coaching and clinical audits in all 17 health facilities. The baseline assessment tool was adapted from the 'health facilities supervision form' from the Nepal PEN manual (2018/2019).^12^ We assessed available resources (medicines and equipment), awareness, and status of PEN Interventions in practice using pre-designed quantitative survey questionnaires. Research Assistants conducted the baseline survey with the health center in-charge. Each session lasted between thirty to forty-five minutes.

*Stakeholders' engagement/meeting:* Following the baseline assessment, a stakeholders' engagement meeting was conducted with municipalities and the health office stakeholders to introduce the study and seek permission for necessary administrative and logistic support; understand stakeholders' perspectives about PEN Interventions and the study; share and discuss the findings of baseline assessment and, reinforce support for intervention implementation and its sustainment. Four municipal health coordinators and one district health coordinator attended the meeting. Research team members took notes of the meeting discussions, which were later considered while developing the intervention.

*Site randomization to control and intervention groups:* The 17 eligible health facilities were randomly assigned to intervention (ni= 9) and control (nc= 8) groups using the RAND function in Excel. To avoid any potential bias during baseline data collection due to the researcher's knowledge of intervention assignment, randomization was done after the baseline assessment was completed.

### Intervention Phase

*Focus Group Discussions (FGDs):* We conducted one FGD with health staff in each intervention site. The FGDs explored a) existing PEN Interventions implementation scenarios in health facilities, b) participants' understandings of PEN Interventions, and c) needs and challenges for effective implementation. All staff who were directly or indirectly engaged in NCD management services at that site participated in the discussions. The first two FGDs were led by the PI and others were led by the RAs.

A total of nine FGDs were conducted (one at each intervention site). On average, each FGD took about 30 minutes. At each FGD, there was 4 to 5 health staff. The FGDs were conducted in the health facility. All FGDs were audio-recorded after the consent of the participants.

The learning from the FGDs were incorporated into the design and implementation of the intervention. Additionally, after the FGD was over, we provided a brief orientation on the importance of peer coaching and clinical audit for effective PEN implementation.

*Selection of a peer coach and a person responsible for clinical audit:* At the end of FGD, in each intervention site, the researchers asked the group to identify a peer coach and clinical audit focal person. We encouraged the selection from amongst PEN-trained staff.

*Intervention:* The intervention primarily consisted of reinforcing peer-coaching and clinical audit processes as indicated in the WHO PEN interventions endorsed by the Nepal Government.^12^ The reinforcement process involved a) pre-peer coaching and clinical audit sessions, b) monthly peer coaching sessions, and c) monthly clinical audits.

#### Pre- Peer-Coaching and Clinical Audit sessions

Pre-peer coaching sessions involved interactions with the selected peer coach and the clinical audit focal person. We discussed the contents of PEN Protocol 1 in detail and demonstrated the mechanisms regarding the different Protocol components, including counseling, diagnosis, management and referral criteria. The session length varied from 30 to 60 minutes and the contents were delivered using a PowerPoint presentation followed by an in-depth discussion. Peer coaches were asked to approach the research assistant if additional support was needed during the peer coaching sessions.

Similarly, with the clinical audit focal persons, we discussed the importance of clinical audit. We provided step-by-step guidance on the clinical audit process and the completion of the audit forms. They were encouraged to conduct the clinical audit every month.

#### Peer coaching sessions

Four peer-coaching sessions were conducted in six months at each intervention site. For the first three months, one session was conducted each month; the last session was conducted three months after the third session. The first peer coaching sessions were provided by the RA based on the FGDs where lack of training and preparedness to deliver peer coaching was voiced by many participants. Subsequent sessions were conducted by the nominated peer coach. The peer coaches did the necessary administrative planning along with the required scheduling and logistics and then coached their peers. The RAs were present during the sessions to encourage and support the peer coach's planning and execution. They also encouraged the peer coaches to motivate other staff to actively interact with the peer coach and to reach out whenever they had confusion.

The sessions were conducted in the health facility, usually in the afternoon (later half of the day), so that usual clinical practice was less impacted. Each session lasted for an hour. The RA evaluated the peer coaching process using a standard PEN checklist.^13^ (Table 1: Supplementary 1) The checklist assessed:

1. Logistics available in health facilities before and on the day of peer coaching,
2. Reporting mechanism (PEN coaching completion and submission to local health supervisor) and discussion after the completion of the session, and,
3. PEN protocol 1 components such as clinical assessment, risk factor assessment, estimating CVD risk, referral, and counseling.^12^

Furthermore, to assess the effectiveness of peer-coaching, we conducted a pre- and post-test among peer-coaching session attendees using pre-designed questions based on PEN protocol 1.^12^ Evaluation was conducted in each four sessions and different sets of questions were used each time. Questions were designed with increasing difficulty levels for subsequent sessions.

Research assistants administered the pre-and post-test questions before and after each session, respectively. The questionnaire was pre-designed and prepared by the research team after a detailed discussion and covered the PEN Protocol 1.^12^ It was scored by the RAs and confirmed by the research team.

#### c) Clinical Audit

We requested that the clinical audit focal person fill out the monthly clinical audit forms. The RA collected the forms on their next visit to the site. The form format was used based on the standard PEN checklist.^13^ We evaluated the filled forms and collected feedback from the focal person every month.

The clinical audit checklist included information, such as patient demographic details, availability of patient follow-up book, diagnosis, risk factors, complications evaluation, 10-year CVD risk score, medicine adherence, and others (Table 2: Supplementary 2).^13^

### Outcomes assessment

The study's key outcome measures included a) implementation outcomes and b) effectiveness outcomes of intervention (peer-coaching and clinical audit).

#### a) Implementation Outcomes:

Implementation Outcomes included: i) acceptability, ii) adoption, iii) feasibility, iv) penetration, v) cost, and vi) sustainability of our peer coaching and clinical audit reinforcement intervention based on Proctors' framework.^9^ These outcomes were assessed in the intervention sites only. Outcomes assessments were done within 6 to 12 months of intervention. In-depth qualitative interviews informed many implementation outcomes, in addition to various quantitative metrics and tools (Table 3: Supplementary 3)^14,15^.

The RA observed and filled the HTN observation checklist (Table 4: Supplementary 4)^13^ during their visit to the respective intervention sites' outpatient departments. The checklist evaluated the service provided to hypertensive patients before and after intervention.

#### b) Effectiveness outcomes of peer-coaching and clinical audit

Peer coaching and clinical audit (pre- and post-intervention) will be evaluated using:

(i) Peer coaching scores, (ii) Internal Clinical audit scores, (iii) Baseline versus Endline scores (Table 5: Supplementary 5)

### Data Analysis:

**Quantitative data:**

Implementation Outcomes: As shown in Table 3 (Supplementary 3), implementation outcomes analysis involved calculating the proportion of the staff trained, the number of audit forms completed, and assessing different checklist scores.

Effectiveness Outcomes: Pre and post-test scores were compared using paired sample t-tests. Before and after scores of the completion of the audit forms were compared in difference of proportion.

**Qualitative data:**

Thematic analysis for analyzing qualitative data that consists of mainly five steps; data familiarization, generating initial codes, searching for themes, reviewing and refining themes, and writing the analysis was adopted.^16^

## DISCUSSION

This paper describes the method used in the development and pilot assessment of peer-coaching and clinical audit reinforcement intervention aimed at improving the implementation of PEN intervention for NCD management in Nepal. Intervention broadly consisted of educating and encouraging health post staff to perform peer-coaching and clinical audits in the routine management of NCDs as indicated by the PEN Intervention. The implementation assessment used a multiple-method approach and was guided by Proctor's Framework.^9^

Nepal, like most LMICs, is facing a rapidly increasing burden of NCDs, threatening the health system capacity and calling for urgent attention to strengthen strategies to tackle them effectively.^17-19^ Despite prioritization and strategies in place to manage the growing burden of NCDs - most strategies fall short due to their ineffective implementation.^19-21^ WHO PEN Interventions is one such strategy. WHO PEN Interventions was introduced in Nepal in 2016 with the main aim of increasing access to NCD-related services in primary health facilities.^12^ At present, primary NCD management using WHO PEN has been rolled out all over Nepal.^22^ However, its implementation remains poor.^4^

The widely advocated mechanisms such as task-shifting and team-based care for managing NCDs in resource-limited settings^1^ are recommended strategies in PEN Interventions. Team-based care, which includes peer coaching and clinical audits, is one of the proposed mechanisms for utilizing WHO PEN implementation.^23^ However, clear evidence and robust studies regarding PEN Intervention implementation and/or effectiveness of mechanisms such as peer coaching/ clinical audit are lacking not only in Nepal but also widely in LMICs.^24-26^ Furthermore, there are no specific guidelines/ detailed steps on how peer coaching and clinical audits should be conducted for effective PEN Intervention implementation.

This study aims to explore if (and how) peer coaching and clinical audit can be reinforced to promote PEN Intervention implementation in Nepal. We developed peer-coaching and clinical audit reinforcement intervention, largely based on PEN Intervention,^1^ but adapting to the context of Nepal.^12^ This study aims to identify the appropriate, cost-effective method of team-based care so that we can achieve the patient-centered primary health care of the current MultiSectoral Action Plan for NCDs in Nepal.^27^ This paper outlines the methods of intervention development and assessment processes.

This method uses strategies and tools outlined in the PEN Intervention-hence, this method will provide insights on not only peer-coaching and clinical audit mechanisms but also the usefulness/applicability of the various tools outlined in PEN Interventions. Similarly, assessment uses robust techniques such as RCT and implementation outcomes evaluation is based on an established implementation framework, namely the Proctor framework.^9^

The tools for the evaluation of the health facility readiness for team-based care, selection of peer coach and person responsible for clinical audit, peer coaching process and its evaluation and clinical audit evaluation process has been compiled. Our intervention will, therefore, provide much-needed information on prospective peer coaching and clinical audit processes, how these methods can be effectively delivered, what could hinder these processes, and if these mechanisms lead to effective PEN Intervention delivery. Additionally, the method will provide valuable insights to properly implement any multidisciplinary efforts toward NCD management in resource-limited settings, including the recently endorsed PEN-plus program.

## Trial Status:

The trial has completed its recruitment.
